# Modulation of the Host Response as a Therapeutic Strategy in Severe Lung Infections

**DOI:** 10.3390/v15071462

**Published:** 2023-06-28

**Authors:** Elyse Latreille, Warren L. Lee

**Affiliations:** 1 Department of Laboratory Medicine and Pathobiology, University of Toronto, Toronto, ON M5S 1A8, Canada; 2 Keenan Research Centre for Biomedical Science, St. Michael’s Hospital, Toronto, ON M5B 1W8, Canada; 3 Department of Medicine, Interdepartmental Division of Critical Care, University of Toronto, Toronto, ON M5B 1T8, Canada; 4 Department of Biochemistry, University of Toronto, Toronto, ON M5S 1A8, Canada

**Keywords:** host response, lung injury, endothelium, vascular stability, inflammation, lung regeneration

## Abstract

Respiratory pathogens such as influenza and SARS-CoV-2 can cause severe lung infections leading to acute respiratory distress syndrome (ARDS). The pathophysiology of ARDS includes an excessive host immune response, lung epithelial and endothelial cell death and loss of the epithelial and endothelial barrier integrity, culminating in pulmonary oedema and respiratory failure. Traditional approaches for the treatment of respiratory infections include drugs that exert direct anti-pathogen effects (e.g., antivirals). However, such agents are typically ineffective or insufficient after the development of ARDS. Modulation of the host response has emerged as a promising alternative therapeutic approach to mitigate damage to the host for the treatment of respiratory infections; in principle, this strategy should also be less susceptible to the development of pathogen resistance. In this review, we discuss different host-targeting strategies against pathogen-induced ARDS. Developing therapeutics that enhance the host response is a pathogen-agnostic approach that will help prepare for the next pandemic.

## 1. Introduction

Infection by respiratory pathogens can lead to severe, life-threatening illness, including sepsis and acute respiratory distress syndrome (ARDS.) Treatment primarily relies on supportive therapies and direct anti-pathogen drugs, such as Tamiflu for influenza, to inhibit replication and dissemination of the virus within the host. However, anti-viral drugs and antibiotics exert a selective pressure on the pathogen, leading to antimicrobial resistance (e.g., adamantanes for influenza) [1]. While the host response to infection varies depending on the pathogen, the downstream effects on the host are often similar. For example, while severe infections with influenza and severe acute respiratory syndrome coronavirus 2 (SARS-CoV-2) cause distinct cytokine profiles, both result in excessive inflammation and a loss of the lung barrier integrity, leading to oedema, tissue damage and poor outcomes [2]. Treating respiratory infections by modulating the host response has emerged as a viable therapeutic approach. The recent coronavirus disease 2019 (COVID-19) pandemic has highlighted the utility of this approach, as treatment with corticosteroids such as dexamethasone significantly improves outcomes [3]. In this review, we discuss potential approaches to treating severe lung infections by modulating the host response. Potential avenues include modulation of host inflammation, increasing endothelial barrier integrity and enhancing regeneration of the damaged epithelium and endothelium post infection.

## 2. Overview of Respiratory Infection and Lung Injury

The human respiratory tract is functionally divided into upper and lower halves: the upper tract includes the tonsils, nasopharynx, oral cavity, oropharynx and larynx while the lower tract includes the trachea, bronchi and lungs [4]. Infections that remain in the upper respiratory tract are associated with less severe symptoms while those that reach the lower respiratory tract are associated with more severe symptoms such as bronchitis, bronchiolitis, pneumonia and ARDS [5]. Both RNA and DNA viruses can infect the human respiratory tract; however, RNA viruses such as respiratory syncytial viruses (RSV), influenza viruses, rhinoviruses, enteroviruses and coronaviruses are more commonly the causative agent of respiratory infections [5,6]. Humans of all ages are susceptible to these pathogens, but children, the elderly and the immunocompromised are the most at risk. The severity of symptoms depends on the causative agent, the environment and the host [4].

The eyes and nose are the primary site of entry for human respiratory viruses. Infection of the lower respiratory tract can occur through direct infection of lung cells of the lower respiratory tract due to preferential binding to sialic acids found deeper in the airways, as is the case for many influenza viruses. Infection of the upper respiratory tract and the subsequent spread to the lower respiratory tract can also occur, as is often the case for coronaviruses [5]. The considerable overlap of clinical presentation often makes it difficult to determine which pathogen is the causative agent of infection without the use of diagnostic tests. Regardless of the virus or the means by which it reaches the lower respiratory tract, infections of the lower respiratory tract culminate in bronchiolitis, bronchitis pneumonia and/or severe life-threatening ARDS. Given its high mortality rate, ARDS is the focus of this review.

ARDS is a severe form of inflammatory lung injury with a mortality rate of 30–40%. Common precipitants include pneumonia, sepsis, aspiration of gastric or oral/oesophageal contents and major trauma [7,8]. It is clinically defined according to the Berlin definition; the first respiratory failure should develop within a week of the clinical insult or presence of new/worsening respiratory symptoms and should not be explainable by cardiac failure. Bilateral opacities should be present upon chest imaging, and these should not be due to nodules, a collapse of the lungs or effusions [8,9,10]. Lung endothelial and epithelial injury as well as diffuse alveolar damage are observed in ARDS patients. An increased alveolar capillary permeability culminates in the onset of pulmonary oedema, with accumulation of fluid, proteins, neutrophils and red blood cells in the alveoli [8]. This translates to hypoxemic respiratory failure. Two points are worth emphasizing. First, ARDS is the final common consequence of many severe lung infections. Second, antimicrobial therapy in the setting of ARDS is often insufficient to prevent death [11]. Thus, optimization of the host response to ameliorate or prevent ARDS represents an important opportunity for therapy and is the focus of our discussion.

## 3. Host Modulation Strategies

Many aspects of the host response can theoretically be targeted to improve pathogen-induced ARDS. These include dampening the inflammatory response, enhancing lung barrier function and enhancing the repair or regeneration of the damaged lungs.

### 3.1. Dampening of the Inflammatory Response

During respiratory virus infection, cytokine production is an important element of the host response. While intended to help clear the infection, excessive cytokine production and uncontrolled inflammation results in tissue injury, ARDS and poor outcomes [12,13,14]. Both influenza and SARS-CoV-2 are associated with a cytokine storm, described as elevated circulating cytokine levels [14]. For example, highly pathogenic avian influenza (H5N1) and the 1918 pandemic influenza (H1N1) have both been shown to induce an early dysregulated innate immune response in the lungs characterized by increased chemokine and cytokine production. This is associated with the pathogenesis and severity of disease of these viruses in macaques [15,16]. Similarly, infection with SARS-CoV-2 has also been shown to greatly increase levels of inflammatory cytokines such as interleukin (IL)-6, IL-2, IL-7, IL-10, granulocyte colony stimulating factor (G-CSF), interferon gamma (IFN-γ), interferon inducible protein 10 (IP-10), tumour necrosis factor alpha (TNF-α) and monocyte chemoattractant protein-1 (MCP-1), which play a role in the pathogenesis of COVID-19 [17]. Influenza virus hemagglutinin protein binds to host cell sialic acid linked to galactose via α2,3 or α2,6 linkages. Human influenza viruses preferentially bind to α2,6-linked sialic acid residues expressed primarily in the upper respiratory tract, while avian influenza viruses bind to α2,3, which are more abundant in the lower respiratory tract [18]. The ability to directly infect the lower respiratory tract may in part explain the higher pathogenicity of avian influenza viruses such as H5N1. Leukocytes and epithelial cells were previously thought to be the primary cell types responsible for the cytokine production upon infection since endothelial cells were not thought to be important contributors to the cytokine storm. However, endothelial cells have been shown to play a role in both direct production of cytokines as well as regulating leukocyte infiltration to the lungs, making them important in both the initiation and amplification of the cytokine storm [14]. Titrating down this inflammatory response to decrease tissue injury without compromising the pathogen clearance is the goal of this approach.

#### 3.1.1. S1P1 Agonism

Critical experiments using the sphingosine 1 phosphate 1 (S1P_1_) receptor agonist CYM-5442 indicate that endothelial cells are important orchestrators of the cytokine storm after lung influenza infection. S1P_1_ is highly expressed on the endothelium and lymphocytes, but is not expressed by the epithelium and other leukocytes. Treatment of influenza-infected mice with CYM-5442 significantly improves survival. This benefit is due to its modulatory effects on the endothelium, as CYM-5442 reduces cytokine and chemokine production in lymphocyte-deficient mice as well as cytokine and chemokine mRNA in lung endothelial cells derived from the mice. The reduction in endothelial chemokine expression also reduces pulmonary infiltration of neutrophils, macrophages and monocytes to the lungs. Importantly, restoring leukocyte infiltration to the lungs does not restore cytokine or chemokine production, suggesting that these infiltrating leukocytes are not important contributors to the initiation of the cytokine storm, but more likely play a role in further exacerbating it. This report played a critical role in establishing that lung endothelial cells are an important potential target to reduce the excessive innate immune inflammatory response [14].

#### 3.1.2. IL-6 Blockade

Although endothelial cells produce many proinflammatory cytokines, IL-6 is of particular interest in viral respiratory infections. IL-6 is one of the main proinflammatory cytokines produced by endothelial cells co-cultured with influenza A (IAV)-infected epithelial cells [19]. While important for host defence, there is abundant evidence that IL-6 can contribute to host injury [20]. Elevated IL-6 is a significant predictor of disease severity of influenza-associated pneumonia [21]. On the other hand, IL-6-deficient mice exhibit increased mortality upon IAV infection [22]. This is likely because its complete deletion interferes with the host’s ability to combat the infection or heal tissues post infection. For instance, IL-6 has been reported to enhance M2 macrophage polarisation for resolution of inflammation and tissue repair [23].

IL-6 has been extensively studied in the context of COVID-19. For example, there are many clinical reports describing elevated cytokines, particularly IL-6, in patients with severe COVID-19 [24,25]. Tocilizumab, an anti-IL-6 receptor antibody, has been shown to be beneficial in a number of COVID-19 clinical trials. In the largest randomized trial of tocilizumab by the RECOVERY group with over 4000 COVID-19 patients, tocilizumab reduced 28-day mortality, increased the probability of discharge and reduced the probability of mechanical ventilation or death in hypoxic patients who were not already receiving mechanical ventilation. Importantly, this benefit is additive to corticosteroid treatment, indicating the blockade of IL-6 is a useful treatment approach for patients with severe COVID-19 [26]. However, this approach has not yet been fully investigated in other severe lung infections.

#### 3.1.3. Corticosteroids

Corticosteroids (typically dexamethasone) are currently recommended for the treatment of severe COVID-19; their binding to the glucocorticoid receptor results in anti-inflammatory effects [27]. However, due to the pleiotropic effects of the glucocorticoid receptor on many signalling pathways, off-target effects such as immunosuppression, metabolic effects, hypertension and osteoporosis are common during long-term treatment [27]. Treatment of SARS-CoV and middle eastern respiratory syndrome coronavirus (MERS-CoV) patients with dexamethasone enhances viral load/delay viral clearance, raising concerns for their use in treatment of COVID-19 [28,29]. In addition, corticosteroid treatment is associated with increased mortality and nosocomial infection in patients with influenza-associated pneumonia and ARDS [30]. However, in patients hospitalized with COVID-19 (RECOVERY group), dexamethasone greatly lowers the 28-day mortality in mechanically ventilated patients or patients requiring respiratory support. As such, corticosteroids became the standard of care in critically ill COVID-19 patients [3,31]. The fact that they are beneficial in severe but not mild cases is further evidence that the host response rather than viral replication is a major determinant of the outcome in the critically ill.

The mechanism of action of dexamethasone in COVID-19 has yet to be fully elucidated. Dexamethasone reduces angiopoietin-2 (Ang-2), intercellular adhesion molecule 1 (ICAM-1), glycocalyx shedding and the soluble receptor for advanced glycation end-products (sRAGE) in critically ill COVID-19 patients, suggesting potential reduction in endothelial damage. Interestingly, markers of epithelial injury such as surfactant protein D do not correlate with C-Reactive protein (CRP), a marker of inflammation. This supports the hypothesis that endothelial dysfunction/injury characterizes severe COVID-19, and that corticosteroids provide a benefit via reducing endothelial injury [32]. The benefit of dexamethasone may also be present via modulating the inflammatory host response (independently of the endothelium) since markers such as CRP, IL-1β, IL-6, IL-8 and TNF-α are significantly reduced by dexamethasone, but endothelial activation, platelet activation, neutrophil extracellular trap (NET) formation and coagulopathy are unaffected [33]. This hypothesis is supported by studies on Syrian hamsters, in which lung injury and mRNA of proinflammatory cytokines such as IL-4, IL-6, IL-10, IL-13, TNF-α and IFN-γ are reduced by dexamethasone treatment 7 days post infection, suggesting its benefit may be due to the inhibition of the cytokine storm [34]. More recently, data have emerged suggesting dexamethasone may regulate cytokine release via decreasing the levels of Kv1.3 calcium channels. Correlation network analyses indicate that dexamethasone decreases calcium ion transport into the cell, calcium-mediated signalling, and lowers the expression of calcium channels such as potassium voltage-gated channel subfamily A member 3 (KCNA3). These findings provide a potential new target for treatment of COVID-19; however, further work is required to elucidate the precise mechanism of action of dexamethasone in COVID-19 [33,35]. Of note, the benefit of dexamethasone does not extend to severe influenza. A meta-analysis suggests that its use is associated with increased mortality [36].

### 3.2. Enhancement of Lung Barrier Function

A loss of integrity of the alveolar–capillary membrane is a pathognomonic feature of ARDS. In the lung microvascular endothelium, adherens junctions and tight junctions connect adjacent endothelial cells. Typically, adherens junctions initiate and maintain cell–cell contact and are composed of cadherins (e.g., vascular endothelial (VE)-cadherin) and catenins (e.g., β-catenin, α-catenin, p120-catenin). Internalization and/or degradation of VE-cadherin leads to increased permeability [37]. Tight junctions are typically composed of occludins, claudins and scaffold proteins [38]. The exact protein composition, organization and number of both types of junctions are variable depending on the tissue and can account for differences in vascular permeability in different organs. In the lungs, the endothelial barrier is relatively tight, but infection can trigger leakage and an increased lung endothelial permeability, leading to oedema and lung damage [39]. As such, enhancing the endothelial barrier integrity in the lungs has emerged as a promising therapeutic strategy for respiratory infections.

#### 3.2.1. Angiopoietin/Tie2 Signalling

Tie2 is a receptor for angiopoietins and is highly expressed by the endothelium. The angiopoietin 1 (Ang1)/Tie2 signalling axis is important in the regulation of endothelial permeability. Binding of Ang1, a strong agonist of Tie2, induces the localisation of Tie2 to cell–cell junctions in monolayers, which promotes endothelial survival, vessel stability and proper endothelial barrier function [40]. Tie2/Ang1 binding also induces vascular protective signalling. For instance, Tie2/Ang1 binding leads to activation of AKT, which causes nuclear exclusion of forkhead box protein O1 (FOXO1) and decreases expression of FOXO1 target genes. This increases expression of genes for vessel stability and decreases expression of genes such as Ang2, which destabilize endothelial monolayers, antagonise Ang1/Tie2 signalling and promote vascular leakage [41,42]. Targeting Tie2 to promote vascular integrity during infection is a promising therapeutic approach. For instance, Vasculotide, a novel peptide Tie2 agonist, improves survival and reduces oedema in a murine model of severe influenza-induced acute lung injury even when administered as late as 72 h after infection. Importantly, the drug has no effect on viral replication and does not impede the recruitment of neutrophils to the lungs [43]. The drug also does not alter circulating or alveolar cytokine levels, highlighting the fact that vascular leakage and the innate immune response can be regulated separately [43,44].

These results have been validated in a model of *S. pneumonia*-induced acute lung injury. Vasculotide promotes endothelial stability and reduces lung permeability and the lung injury score and does not affect neutrophil recruitment to the lungs or cytokine production. Further mechanistic work in endothelial cells confirmed vasculotide treatment leads to phosphorylation of Tie2, prevents gap formation, reduces stress fibre formation and increases transcellular electrical resistance upon lipopolysaccharide (LPS) or pneumococcal endotoxin pneumolysin treatment [45]. Similar findings have also been reported in infected mice undergoing mechanical ventilation [46].

Other approaches to manipulating Tie2 signalling have also shown promising results. Ang2 antagonises the Ang1/Tie2 pathway and is released in response to inflammatory stimuli such as infection, resulting in a vascular leak. Since Ang1 levels remain relatively stable and Ang2 levels rise during infection, an Ang2 blockade presents an alternative approach to increase the barrier integrity via the Tie2 signalling axis. Indeed, treatment of influenza-infected mice with L1-7, a peptide–antibody inhibitor of Ang2, reduced hypoxia, oedema, bronchoalveolar lavage (BAL) protein and alveolar thickening, and improved survival [47].

#### 3.2.2. Apelin

Apelin is a ligand for the G protein-coupled receptor angiotensin II type 1 receptor-related protein (APJ). The apelin APJ axis was induced in a rat model of oleic acid-induced ARDS. Agonism of apelin–APJ signalling reduces lung pathology, while its inhibition worsens lung pathology, leakage, oedema and hypoxemia [48]. In vitro, treatment of human umbilical vein endothelial cells (HUVECs) with apelin prevents the vascular endothelial growth factor (VEGF) and histamine-mediated loss of VE-cadherin, gap formation, permeability and stress fibre formation. Furthermore, apelin prevents VEGF-induced internalisation of VE-cadherin, without altering the total VE-cadherin levels [49]. Similarly, in mice with LPS-induced lung injury, administration of apelin-13 alleviates histological changes and decreases the wet to dry ratio and levels of IL-6, IL-1β and TNF-α. Importantly, apelin increases VE-cadherin levels and reduces its phosphorylation, preventing its internalisation [50].

The protective mechanism of apelin has been linked to friend leukemia integration 1 transcription factor (FLI-1), a transcription factor that regulates expression of genes such as VEGF, VE-cadherin, S1P and other genes involved in vascular maintenance. FLI-1 expression is downregulated in mice in the first 24 h post administration of LPS, accompanied by decreased VE-cadherin and increased VEGF and SRC, which disrupts adherens junctions. Apelin treatment reverses these effects and decreases internalization of VE-cadherin, thus increasing barrier stability. Inhibition of FLI-1 reduces the benefit of apelin treatment [51]. As such, the apelin-APR signalling axis may be an important therapeutic target to increase junctional integrity and endothelial barrier stability during acute lung injury caused by infections.

#### 3.2.3. Sirtuins

Sirtuins (SIRTs) are histone deacetylases and play a protective role against aging by regulating metabolism, mitochondrial maintenance, DNA repair, telomere stability and autophagy. Their action requires nicotinamide adenine dinucleotide (NAD+). Their expression decreases with age as well as in certain pathologies such as fibrosis [52]. In murine models of LPS-induced lung injury, sirtuins have been shown to have beneficial roles. In particular, SIRT1 has been shown to directly reduce lung endothelial permeability. Intratracheal administration of LPS decreases expression of SIRT1 as well as the tight junction proteins occludin, claudin-5, TJP1 and TJP2 in the mouse lungs; this is accompanied by increased oedema and lung injury [53]. Increased ubiquitination of SIRT1 upon LPS treatment of endothelial cells has been reported, suggesting SIRT1 may be lost via degradation [54]. Administration of a SIRT1 activator to mice can reduce lung injury and permeability, potentially due to its ability to increase tight junction protein expression [53,54]. In vitro, SIRT1 activation increases transendothelial electrical resistance and reduces the flux of fluorescein-labelled dextran across monolayers of primary human pulmonary vascular cells [53,54].

In addition to preserving tight junctions, SIRT1 has also been shown to modulate VE-cadherin. LPS or a cecal ligation puncture (CLP) reduces VE-cadherin expression and lowers its association with β-catenin, which can be prevented by activation of SIRT1. Indeed, SIRT1 activation reduces stress fibre formation, prevents disruption of VE-cadherin distribution and prevents downregulation of β-catenin, another important protein involved in endothelial adherens junctions during LPS treatment [53,54].

Preliminary mechanistic work has also identified SIRT1 as a regulator of oxidative stress/inflammation. SIRT1 prevents the loss of the detoxifying enzyme SOD 2 and reduces NOX expression, leading to reduced ROS production upon LPS treatment. These findings suggest SIRT1 may also play a role in maintaining the endothelial barrier in response to oxidative stress induced by infection [54].

Beyond SIRT1, other sirtuins were shown to be beneficial in lung injury. Overexpression of SIRT3 increases VE-cadherin levels as well as its association with β-catenin upon LPS treatment. In a mouse model of sepsis-induced acute lung injury, SIRT3 maintains the endothelial barrier via modulation of the VE-cadherin/β-catenin interaction and preservation of adherens junctions. SIRT3 knockout (KO)-mice challenged with LPS or CLP exhibit a worsened lung injury score, decreased VE-cadherin and increased IL-6 and TNF-α [55].

Recent work has also identified SIRT7 as a potential regulator of vascular permeability in pulmonary endothelial cells. Silencing of SIRT7 in LPS-treated endothelial cells increases inflammatory cytokines such as IL-6 and IL-8 as well as adhesion molecules such as VCAM-1 and ICAM-1. Junctional proteins such as VE-cadherin are also decreased, accompanied by increased permeability and F-actin stress fibre formation [52]. While studies of sirtuins in viral models are limited, these studies suggest that activating or increasing sirtuin expression during infection-induced lung injury may increase adherens and/or the tight junctional integrity and decrease endothelial permeability.

## 4. Regeneration of the Damaged Lung

While manipulation of cell–cell junctions may decrease endothelial permeability, this approach may not be feasible when endothelial cells have died and gaps in the monolayer already exist. It may also be ineffective in the setting of epithelial cell death. Infection with respiratory pathogens leads to both direct lung damage due to pathogen-induced cell death, as well as indirect lung damage caused by the host response, for example, by the cytokine storm and inflammation. As such, elucidation of the mechanisms orchestrating epithelial and endothelial repair is very important.

Epithelial cells of the airway are exposed to the outside world including particulates and inhaled pathogens. There are region-specific stem cell populations to help maintain and repair the lung epithelium following injury. Two types of epithelial cells are present in the alveolus: alveolar type I and alveolar type II cells (AT1 and AT2, respectively). The majority of the surface area of alveoli (approximately 95%) is composed by the large AT1 cells, where gas exchange occurs [56]. AT1 cells are thought of as being terminally differentiated with little to no proliferation. The remaining 5% of alveolar epithelial cells are AT2 cells, which are smaller, produce pulmonary surfactant and have self-renewing and progenitor properties [57,58]. Overall, the lung has numerous types of stem/progenitor cells, the most studied being tracheal basal cells, airway club cells and AT2 cells [59]. The proliferation of AT2 cells contributes to alveolar repair following injury by replenishing lost AT2 cells and differentiating into AT1 cells to regenerate the alveolus [57,58]. Regulating endogenous epithelial stem/progenitor cells is a potential approach to promote regeneration of the damaged lung epithelium. This is a highly active area of research [60], as reviewed in [58].

By comparison, the role of endothelial repair and regeneration has been less well studied. The realization that local (rather than marrow-derived) endothelial progenitor cells orchestrate vascular repair has raised interest in the notion of enhancing endothelial regeneration by activating so-called intrinsic endothelial regeneration mechanisms; the recent literature has highlighted the role of transcription factors that may be involved.

### 4.1. Sox17

The transcription factor SRY (Sex Determining Region Y)-Box 17 (Sox-17) plays many roles, including developmental regulation in endothelial and hematopoietic cells, arterial integrity, angiogenesis in tumours, conversion of fibroblasts to reparative endothelial cells and more. Importantly, Sox17 plays a role in restoring endothelial integrity. In a model of LPS-induced lung injury, the endothelial cell population is greatly reduced 1 day post LPS injection, and restored by day 7. Compared to wild-type (WT) mice, Sox17 KO mice exhibit reduced lung endothelial cells 5 days post LPS. Importantly, restoring Sox17 in KO mice by liposomal delivery 3 h after injury increases endothelial proliferation, restores lung endothelial barrier function and improves survival. Moreover, induction of Sox17 expression increases Cyclin E1 expression, suggesting induction of endothelial proliferation and regeneration following endotoxin-induced injury [61]. 

After LPS-induced lung injury, two distinct subpopulations of endothelial cells have been identified: one enriched for expression of immune response genes (immuneEC) and one with increased expression of vascular development genes (devEC). During early inflammation (6 h and 24 h post LPS), these two populations are present and have either an immune (interferon regulatory factor 7, IRF7+) or developmental (Sox17+) phenotype (immuneEC or DevEC, respectively). In this model, endothelial regeneration and repair typically begin by 2 days post LPS; there are reduced immuneEC (IRF7+) cells and increased DevECs (Sox17+) during recovery [62]. Interestingly, in stimulated endothelial cells, overexpression of Sox17 prevents IL-1β and TNF-α expression, while Sox17 knockdown enhances their production, suggesting that Sox17 might also prevent excessive endothelial inflammation. The endothelial subpopulations have also been identified in lungs of non-human primates infected with SARS-CoV-2 as well as in mice infected with influenza [62].

### 4.2. FoxM1

Forkhead box protein M1 (FOXM1) is a transcription factor expressed by proliferating cells. It controls progression of the cell cycle into G1/S and G2/M (DNA replication and mitosis, respectively) by controlling expression of S-phase kinase-associated protein 2 (SKP2), cyclin-dependant kinase subunit 1, cyclin B1, CDC25B, CDC25C phosphatases, aurora kinase and others. It has emerged as a potentially useful target for regeneration of the lungs following injury [63]. Expression of FOXM1 can be induced in lung epithelial cells for alveolar repair. For example, intratracheal administration of *P. aeruginosa* in mice increases FOXM1 expression in type II alveolar epithelial cells. These cells can function as progenitor cells for alveolar type I cells, resulting in alveolar epithelial barrier repair. Indeed, FOXM1 was preferentially expressed in type II alveolar cells expressing progenitor cell marker stem cell antigen-1 (SCA1). In mice lacking type II cell expression of FOXM1, type II cells exhibit decreased proliferation and trans-differentiation into alveolar type I cells accompanied by defective barrier repair post injury. These findings indicate the importance of FOXM1 in the proliferation and transition of epithelial type II cells into type I cells [64]. 

Inflammatory stimuli such as LPS and TNF-α have also been shown to increase FOXM1 expression in murine and human endothelial cells. The importance of FOXM1 is implied by knockdown studies, as depletion of FOXM1 in human endothelial cells disrupts cell cycle progression. Increased FOXM1 expression is also detectable in the lungs of LPS-challenged mice at 2 days post challenge and peaks by the 3rd day, which coincides with resolution of lung injury. After exposure to LPS, mice lacking endothelial FOXM1 exhibit an increased vascular permeability, increased p27kip1 (cyclin inhibitor) and decreased expression of cyclin B1 and cdc25C; importantly, the animals also exhibit decreased survival. These findings suggest that impairment of FOXM1 activation during inflammatory vascular lung injury may prevent regeneration of the endothelium [65].

While the role of endothelial FOXM1 has yet to be investigated in viral lung injury, inducing FOXM1 expression may be a promising approach.

### 4.3. COUP-TF2

In a non-fatal murine model of influenza (PR8) infection, lung endothelial cells are reduced in number 10 and 19 days post infection, and then increase as the animals recover 27 days post infection. Endothelial proliferation is evident by day 12, and surviving endothelial cells are responsible for vascular regeneration. Chicken ovalbumin upstream promoter-transcription factor 2 (COUP-TF2) was identified as a transcription factor essential for lung endothelial proliferation and vascular regeneration. Knockout of endothelial COUP-TF2 reduces survival of influenza-infected mice; proliferation and angiogenesis are reduced in endothelial cells of knockout mice at 10 days while the lungs of surviving KO mice 25 days post infection display worsened inflammation and injury. Interestingly, levels of COUP-TF2 decrease upon influenza infection in WT mice, despite its importance in vascular repair. Cytokines produced during influenza infection such as IL-1β and TNF-α induce NF-κB, which downregulates COUP-TF2. The mechanism by which COUP-TF2 induces endothelial proliferation involves its binding to the CCDN1 (cyclin D1) promoter. In COUP-TF2 knockout in cells, CCDN1 expression and proliferation are reduced. COUP-TF2 also binds the promoter of neuropilin-1 and promotes VEGFA signalling, contributing to angiogenesis and migration [66]. The activation of COUP-TF2 may have other beneficial effects in the context of the infected lungs as a loss of COUP-TF2 (also known as Nr2f2) during influenza infection contributes to endothelial dysfunction. Silencing of COUP-TF2 in endothelial cells causes inflammation and increases reactive oxygen species production, both of which can be detrimental when excessive during infections. Notably, in COUP-TF2-deficient cells, IFN-α signalling is a strongly activated inflammatory pathway, accompanied by an increased signal transducer and activator of transcription 1 (STAT1) expression and activation [67]. Altogether, this provides strong evidence that stabilizing COUP-TF2 may be a potential approach to enhance recovery from severe lung injury.

### 4.4. ATF3

As with the lung epithelium, different subtypes of lung endothelial capillary cells exist. The so-called CAP1 (gCAP, general capillary) endothelial cell is thought to be a dominant source of endothelial proliferation after elastase-induced injury [68] while the CAP2 (aCAP, aerocyte capillary) cell is generated late in embryonic lung development [69]. The mechanisms involved in the initiation of CAP1 endothelial cell proliferation are incompletely understood, as is the level of contribution of CAP1s to endothelial regeneration post injury. Activating transcription factor 3 (ATF3) has been identified as being differentially expressed in CAP1 and CAP2 endothelial cells. ATF3 expression is elevated in a subset of CAP1 cells, termed CAP1_Bs. This population of cells is enriched 14 days post influenza-induced lung injury and is thought to be important for endothelial regeneration. In mice, after influenza-induced lung injury, ATF3 expression increases in lung endothelial cells, but not other proliferating cell types such as lung epithelial AT2 cells. However, it is not required for endothelial proliferation as only 40–60% of proliferating endothelial cells post injury express ATF3. While knockout of endothelial ATF3 does not affect alveolar inflammation and tissue damage at 21 days post influenza infection, it does cause formation of abnormal alveolar structures characterized by enlarged airspaces and defective alveolar regeneration. A loss of ATF3 also reduces expression of Fgfr1, Notch4, Wnt, Map3k6 and other proteins important in signalling associated with cell proliferation, differentiation, angiogenesis and cytoskeletal organization. Furthermore, knockout of endothelial ATF3 reduces the number of proliferating lung cells and causes increased apoptosis post infection [69]. As such, increasing ATF3 or its downstream pathways may be a therapeutic strategy to treat viral lung injury.

### 4.5. S1P

In addition to regulating the cytokine storm and permeability, manipulating S1P signalling may activate endothelial regeneration and lung repair. Using inducible S1PR1 knockout mice and S1PR1-GFP reporter mice, one group observed that intraperitoneal injection of LPS into mice induces the expression of S1PR1 on the majority of lung endothelial cells. The generation of this population was required for lung vascular repair after injury. Bone marrow transplant studies of S1PR1+ endothelial cells into LPS-exposed mice lacking endothelial S1PR1 leads to integration and regeneration of the endothelium. This effect requires STAT3, as inhibition of STAT3 prevents LPS-induced generation of the S1PR1 endothelial cell population and persistence of lung injury. STAT3 induces transcription of sphingosine kinase (SPHK1) and the S1P transporter SPNS2, important for the generation and transportation of S1P, respectively. As such, therapeutics inducing this S1PR1+ population of endothelial cells could increase endothelial regeneration and potentially improve outcomes following pathogen-induced lung injury [70].

## 5. Challenges in Host Modulation

Modulation of the host response to improve pathogen-induced ARDS is both promising and challenging. Traditionally, host-directed therapies have targeted immune cells to increase immunity or to block specific cytokines associated with pathology of a given infection. Other host-directed therapies target pathways required for pathogen survival and replication, which are more pathogen specific. Such approaches are beyond the scope of this review, and are reviewed elsewhere [71,72,73]. In this review, we have emphasized endothelial targeting approaches, as the endothelium is often overlooked despite playing an important role in pathogen-induced lung injury. However, many potential host-modulating compounds have been found to be ineffective (and occasionally harmful) for the treatment of ARDS, including administration of exogenous surfactant, anti-oxidants, non-steroidal anti-inflammatory drugs and others [74]. Many strategies appear promising at the preclinical level only to fail in human clinical trials. The poor translation of potential therapies from pre-clinical to clinical models can be attributed to numerous factors, such as species-specific differences between humans and mice and the simplistic nature of pre-clinical models compared to the complexity of critically ill human patients [75,76]. Patients often have comorbidities such as diabetes mellitus, chronic obstructive pulmonary disease or cancer, while mice used for studies are relatively young and healthy. Pre-clinical studies are often of a prophylactic nature (i.e., administration of a compound before the infection), which is less relevant to the clinical setting. It is also likely that the benefit of specific host-modulating approaches will depend on the inciting pathogen: the utility of corticosteroids for COVID-19 but not for severe influenza is an example. Better characterization of ARDS generally and in response to specific pathogens is likely to be necessary for appropriate targeting and timing of treatment.

Nonetheless, there is reason for optimism. Human clinical trial data (e.g., dexamethasone and tocilizumab for COVID-19) strongly support the notion of modulation of the host inflammatory response in the setting of severe lung infections [77]. Furthermore, much recent progress has been made to understand how lung epithelial and endothelial repair and regeneration are regulated. These advances suggest that it may one day be possible to heal the injured lungs in ARDS. This is fertile ground for ongoing research as we recover from this pandemic and prepare for the next.
